# Selective IT neurons are selective along many dimensions

**DOI:** 10.1152/jn.01151.2015

**Published:** 2016-01-28

**Authors:** Kalathupiriyan A. Zhivago, S. P. Arun

**Affiliations:** Centre for Neuroscience, Indian Institute of Science, Bangalore, India

**Keywords:** inferotemporal cortex, monkey, object recognition, selectivity, shape coding

## Abstract

Our visual abilities are unsurpassed because of a sophisticated code for objects located in the inferior temporal (IT) cortex. This code has remained a mystery because IT neurons show extremely diverse shape selectivity with no apparent organizing principle. Here, we show that there is an intrinsic component to selectivity in IT neurons. We tested IT neurons on distinct shapes and their parametric variations and asked whether neurons selective along one dimension were also selective along others. Selective neurons responded to fewer shapes and were narrowly tuned to local variations of these shapes, both along arbitrary morph lines and along variations in size, position, or orientation. For a subset of neurons, selective neurons were selective for both shape and texture. Finally, selective neurons were also more invariant in that they preserved their shape preferences across changes in size, position, and orientation. These observations indicate that there is an intrinsic constraint on the sharpness of tuning for the features coded by each IT neuron, making it always sharply tuned or always broadly tuned along all dimensions. We speculate that this may be an organizing principle throughout visual cortex.

in primates, object recognition depends critically on the inferior temporal (IT) cortex. Understanding how IT neurons represent objects has been difficult because they show extremely diverse patterns of selectivity. Two distinct observations have been made regarding selectivity in IT neurons: *1*) when tested with distinct stimulus sets (typically, natural stimuli), some neurons show dramatic “sparse” responses i.e., respond to very few stimuli, whereas other cells show distributed responses (Gochin et al. 1994; Tamura and Tanaka 2001); and *2*) when IT neurons have been tested using stimuli that vary gradually along parametric dimensions, some cells show narrow tuning, and others show broad tuning (Op de Beeck et al. 2001 Zoccolan et al. 2007). Surprisingly, these two observations have never been made on the same set of neurons. This is an important question because these two observations can be linked in two ways, each with different implications (Fig. 1).

**Fig. 1.**
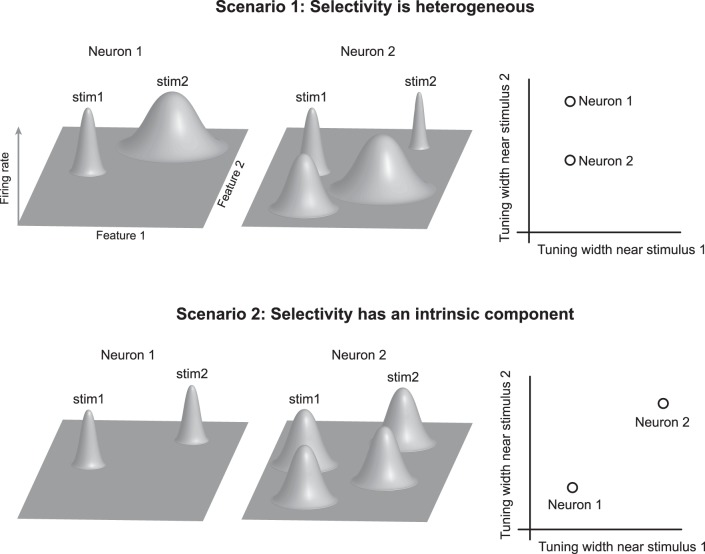
Possible relationships between selectivity across shape space. Consider 2 inferior temporal (IT) neurons: the first neuron is a sparse firing neuron that responds to 2 distinct stimuli (*left*). The second neuron is a distributed firing neuron that responds to 4 distinct stimuli (*middle*). Each row represents a scenario that shows how each neuron might respond to small variations in the neighborhood of its preferred stimuli. *Top* row: Scenario 1: selectivity is heterogeneous. Neuron 1 is sharply tuned to changes in stimulus 1 (stim1) and broadly tuned to variations around stimulus 2, whereas neuron 2 is sharply tuned around both stimuli. According to this scenario, the tuning of a neuron depends on how well stimuli match the preferred features of the neuron and is therefore heterogeneous with no overall constraint. This predicts no correlation across neurons between their tuning widths in the neighborhood of the 2 stimuli. *Bottom* row: Scenario 2: selectivity has an intrinsic component. Neuron 1 shows consistently sharp tuning to variations around all stimuli, whereas neuron 2 shows consistently broad tuning to variations around both stimuli. In other words, selective neurons respond to fewer stimuli and are narrowly tuned in the local neighborhood of each stimulus, whereas less-selective neurons respond to many stimuli and are broadly tuned to local variations of each stimulus. This predicts a positive correlation across neurons between their tuning widths across stimuli. This possibility imposes no constraint on the features preferred by each neuron but rather, constrains the sharpness of tuning in the neighborhood of each feature.

Consider, for instance, two IT neurons depicted in Fig. 1. The first neuron is the classic sparse IT neuron that responds to only two stimuli, whereas the second neuron is a more-distributed firing neuron that responds to several stimuli. How would these neurons respond to small parametric variations of these stimuli? The first possibility is that selectivity is heterogeneous: how fast the firing rate changes to local variations around a stimulus depends on how well these variations match the preferred features of the neuron. In other words, tuning width is unconstrained and heterogeneous. This possibility predicts no systematic correlation across neurons between tuning width near one stimulus and tuning width near another.

A second, more intriguing possibility is that there is an intrinsic, dimensionality-reducing constraint on shape tuning for each neuron. In other words, the first neuron responds to fewer stimuli and is narrowly tuned in the local neighborhood of each stimulus, whereas the second neuron responds to many stimuli and is broadly tuned to local variations. This possibility predicts a systematic correlation across neurons, whereby tuning width near one stimulus predicts tuning width near another. This possibility imposes no constraint on the features preferred by each neuron but rather, constrains the sharpness of tuning in the neighborhood of each feature.

What evidence do we have in favor of each possibility? The first one (that local selectivity is heterogeneous) is consistent with a series of influential studies in which IT neurons were tested on parametrically varying shapes (Brincat and Connor 2004; Hung et al. 2012; Yamane et al. 2008). According to these studies, the response of a neuron to local variations around a shape depends on how its feature tuning is modulated by these variations. However, these studies do not provide explicit evidence for or against this possibility because they have not compared tuning widths across shapes or across model subunits. The second possibility (that selectivity has an intrinsic component) is supported by evidence from early visual areas, where tuning bandwidth of orientation and spatial frequency is correlated (De Valois et al. 1982; Stevens 2004; Xing et al. 2004). It is also supported by the finding that highly selective IT neurons are less tolerant to changes in size, position, and contrast (Zoccolan et al. 2007). Although this has been interpreted as a tradeoff between selectivity and invariance, it is consistent with the more-general alternative that highly selective IT neurons are highly selective along any stimulus variation. These two possibilities can be distinguished by measuring neuronal tuning to small variations of individual shapes and their identity-preserving transformations.

We investigated these issues by recording neural responses in IT of two macaque monkeys performing a fixation task. The stimuli comprised a reference set of eight distinct silhouette shapes to allow for easy manipulation. Each stimulus was varied gradually by morphing it smoothly into another stimulus or by systematically changing its size, position, or orientation. Our main finding is that each IT neuron shows a characteristic sharp or broad tuning for all stimulus variations, suggesting that it has an intrinsic tendency to be sharply or broadly tuned. To investigate whether this result holds for dimensions other than shape, we tested a subset of neurons on a set of textures and a set of shapes. Here too, we found that neurons that were highly selective for texture were also highly selective for shape.

## MATERIALS AND METHODS

We used standard procedures for surgical preparation, behavioral training, and neurophysiological recordings, with details as described previously (Zhivago and Arun 2014). Here, we review only the details most relevant to this study. All experiments were performed according to a protocol approved by the Institutional Animal Ethics Committee of the Indian Institute of Science (Bangalore, India) and by the Committee for the Purpose of Control and Supervision of Experiments of Animals, Government of India.

### 

#### Neurophysiology.

Two adult male macaque monkeys (*Macacca radiata*; laboratory designations *Ka* and *Sa*; aged ∼7 yr) were used in this study. Each animal was surgically implanted with a headpost and a recording chamber, positioned using structural MRI, to be over the anterior portion of the left IT cortex. The recording sites were subsequently verified using structural MRI to be in the anterior inferotemporal cortex. The recorded sites were centered on anterior 14 mm and lateral 13 mm in monkey *Ka* and anterior 19 mm and lateral 15 mm in monkey *Sa* relative to the interaural plane. In monkey *Ka*, the recording chamber was positioned over anterior +19 mm but was tilted by 12° posteriorly and 7° laterally, making the effective recording location anterior +14 mm. Eye movements were monitored using an infrared eye tracker (ETL-250; Iscan, Woburn, MA). Stimuli were displayed on a 120-Hz liquid crystal display monitor (VX2268wm; ViewSonic, Brea, CA) under the control of a computer running Cortex (National Institute of Mental Health, National Institutes of Health, Bethesda, MD). On each day of recording, a 24-channel microelectrode (U-Probe; Plexon, Dallas, TX; 100 μm intercontact spacing along the shank) was inserted through a stainless-steel guide tube and advanced until phasic visual responses were observed on at least one channel. The wide-band signal was stored and processed offline into individual spike trains using commercial spike-sorting software (Offline Sorter; Plexon). Waveforms that formed distinct clusters in principal component analyses were sorted as single units, and those with multiple inseparable spikes were sorted as multiunits. We used only single units with extremely clear isolation for the purposes of this study. In all, we recorded from a total of 49 sites (24 channels/site; 27 sites from *Ka* and 22 from *Sa*), which yielded a total of 366 well-isolated single units. Rarely did these single units include the same action potentials recorded on neighboring channels. These instances were detected by finding cross-correlograms with strong peaks at zero lag. In each strongly correlated pair of units, the unit with the smaller amplitude waveform was removed from further analyses. We identified a total of 155 single units that were visually responsive from this set. Because our goal was to measure selectivity of neurons on both smooth morphs and variations in size, position, and orientation, we selected a subset of 99 units (54 from *Ka* and 45 from *Sa*) that were visually responsive to both smooth morphs as well as to size, position, and orientation variations. However, our results were unchanged, even when repeated on the entire set of visually responsive cells, except that the correlations were weaker overall, due to the inclusion of cells that responded to the morphed stimuli but not the size/position/orientation variations or vice versa. We also confirmed that our results were qualitatively similar for the cells from each monkey considered separately.

#### Behavior.

Animals were trained to perform a fixation task. In each trial, a red fixation dot was shown at the center of the screen, and the monkey was required to look at the dot within 500 ms of its appearance. Following this, eight stimuli were presented for 200 ms, with an interstimulus interval of 200 ms. The animal received a juice reward for successfully maintaining its gaze within a 3° window. Error trials were repeated after a random number of other trials. Both monkeys performed this task at high accuracy for most sessions (average percent correct: *Ka*, 87%; *Sa*, 83%). Although our fixation window was large, a post hoc analysis revealed that both monkeys maintained their gaze close to the fixation dot (average SD across both monkeys: 0.25° and 0.33° along the horizontal and vertical, respectively).

#### Stimuli.

Each neuron was tested on a total of 116 stimuli, each presented eight times to obtain a reliable estimate of firing rate. The reference set consisted of eight silhouette shapes, presented as white against a black background: camel, cat, face, lamp, bird, jeep, tree, and jug (measuring 3° along the longer dimension). We chose silhouettes to permit easy morphing. To create arbitrary parametric variations for each stimulus, we grouped the eight shapes into four pairs and created intermediate-morphed shapes using commercial software (3DS MAX; Autodesk, San Rafael, CA). Each shape in a pair was created using nonuniform rational B-spline (NURBS) curves with the same number of control points matched to corresponding control points on the other shape. A total of nine intermediate morphs was generated between each shape pair. Thus there was a total of 11 stimuli along a particular morph line and 4 morph lines in all, resulting in a total of 44 stimuli.

To create further parametric morphs along identity-preserving transformations, we varied the size, position, and orientation of each shape in the reference set by three levels each. Thus each shape in the reference set was presented at a size of 3° (the reference size), 1.5°, 4.5°, and 6.0°. For position, each shape was tested at 0° (the reference position), 1.5°, 3.0°, and 4.5° in the right visual field (i.e., on the contralateral side). For orientation, each shape was tested at 0° (the reference orientation), 30°, 60°, and 90° rotations clockwise. In all, there were nine additional variations of each of the eight stimuli (3 transformations × 3 levels). This resulted in a total of 72 stimuli with varying size, position, or orientation (8 shapes × 9 variations/shape).

#### Trial design.

Stimuli were presented in a pseudorandom order in each trial with the constraint that only one transformed version of a given object appeared in a trial. Each stimulus was repeated 8 times, and the entire experiment consisted of 116 correct trials.

#### Calculation of sparseness.

We measured selectivity for each neuron using a measure of sparseness used in previous studies (Vinje and Gallant 2000; Zoccolan et al. 2007). For a neuron with responses *r*_*1*_ *r*_*2*_ *r*_*3*_ … *r*_*n*_, where *n* is the number of stimuli tested, the sparseness is defined as follows: S = [1 − (∑*r*_i_/*n*)^2^/(∑*r*_i_^2^/*n*)]/(1 − 1/*n*). This measure ranges from zero for equal firing to all stimuli to one for exclusive firing to only one stimulus in a set. For this and all other analyses, we calculated the firing rate of each neuron in a 50- to 300-ms window after stimulus onset.

#### Absolute tolerance.

Following previous studies (Zoccolan et al. 2007), we defined absolute tolerance for a neuron as the extent to which its response is modulated by variations in size, position, or orientation. For each neuron, we calculated its absolute size tolerance for a given shape as 1 − (R_max_ − R_min_)/(R_max_ + R_min_), where R_max_ is the maximum firing rate of the neuron across all sizes, and R_min_ is the minimum firing rate across all sizes. This tolerance value ranges from zero (when R_min_ = 0; i.e., the neuron shows both 0 and non-0 responses) to one (when R_max_ = R_min_; i.e., a neuron shows no variation in firing rate). This tolerance value was averaged across all eight shapes to obtain the absolute size tolerance. We proceeded similarly to calculate absolute position and orientation tolerance.

#### Relative tolerance.

We defined relative size tolerance as the degree to which a neuron preserves its shape preference across changes in size. This was measured by calculating the correlation coefficient between the firing rates evoked by the shapes in the reference set at each pair of sizes and averaging this correlation across all six size pairs. This measure can range from −1 (for a neuron that reverses its shape preference from one size to another) to 1 (for a neuron that maintains its shape preference across all sizes). We proceeded similarly to calculate the relative tolerance for position and orientation.

## RESULTS

### 

#### Are IT neurons selective along many dimensions?

We recorded the responses of 99 IT neurons from 2 monkeys (54 from *Ka* and 45 from *Sa*) performing a fixation task. Each neuron was tested with a fixed reference set consisting of eight distinct silhouette shapes. Each shape was then parametrically modified either by morphing it smoothly into another shape or by systematically varying its retinal size, position, or overall orientation. To create smooth morphs, we paired each shape with another shape and generated continuous intermediate morphs (see materials and methods).

**Fig. 2.**
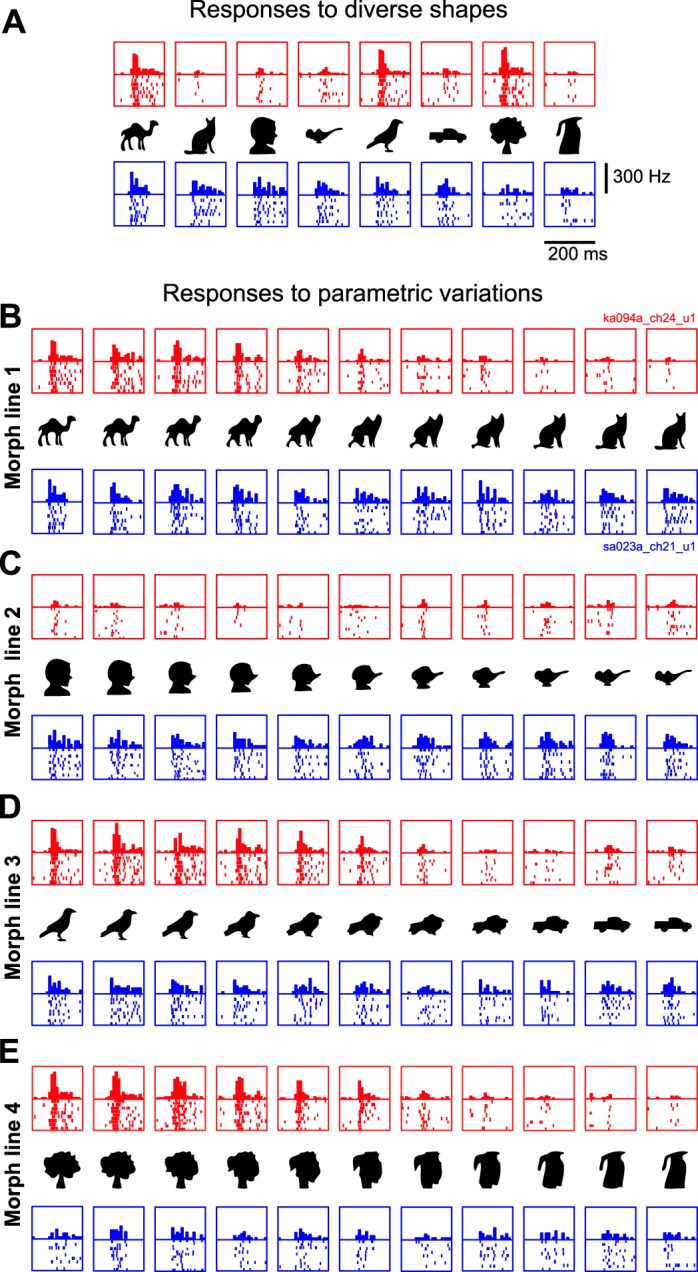
Example responses to the reference set (*A*) and to morphed variations (*B–E*). The first neuron (red rasters) fires sparsely to shapes in the reference set and has sharp tuning to smooth parametric variations of each stimulus. The second neuron (blue rasters) shows graded firing to all stimuli in the reference set and also shows broad tuning to smooth parametric variations. Each box depicts the individual spikes elicited by that stimulus and the histogram with the average firing rate across trials in bins of 10 ms each.

The responses of two example IT neurons to the reference set and the parametric variations are shown in Fig. 2. The first neuron fired sparsely to the diverse set of shapes, whereas the second neuron exhibited graded responses to all of the reference shapes (Fig. 2*A*). When tested on parametric variations of each stimulus (Fig. 2, *B–E*), these two neurons showed very consistent patterns of firing: the first neuron showed consistently narrow tuning along every morph line, whereas the second neuron showed consistently broad tuning. These two neurons exemplify the trends we observed in the recorded population.

**Fig. 3.**
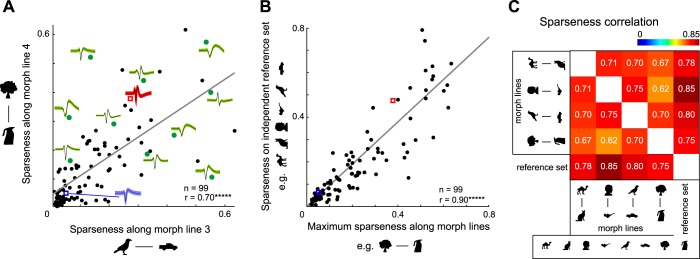
Highly selective neurons are selective along many stimulus variations. *A*: sparseness along morph line 4 plotted against sparseness along morph line 3 for each neuron. Action potential waveforms (black lines within green, red, and blue shaded areas indicate means; green shaded areas indicate SD) are shown for a subset of neurons (green dots) to avoid crowding. The red and blue squares correspond to the red and blue example neurons shown in Fig. 2. *B*: sparseness on the reference set (excluding the morph pair that gave maximum sparseness) plotted against maximum morph line sparseness across neurons with conventions as before. *C*: matrix of pairwise correlations between reference set sparseness and sparseness along each individual morph line. All correlations were statistically significant (*****P < 0.000005).

To quantify neuronal tuning for shapes, we calculated a measure of sparseness (Vinje and Gallant 2000; Zoccolan et al. 2007) based on the firing rate of each neuron in a 50- to 300-ms window after stimulus onset. This measure ranges from zero for a neuron with identical responses to all stimuli to one if it responds to only one stimulus in a set. For each neuron, we measured sparseness on the reference set of diverse stimuli and along each morph line and asked whether these measures are related across neurons. Although, in principle, the sparseness values along two morph lines can be compared in magnitude, this may not be a meaningful comparison because the steps along the morph lines are not equated in any meaningful way. Rather, our goal was to ask whether a neuron that is sharply tuned along one morph line would be sharply tuned along another. Upon performing this comparison, we observed striking correlations: sparseness along one morph line was strongly correlated with sparseness along another (*r* = 0.70, *P* < 0.000005, between morph lines 3 and 4; Fig. 3*A*). To compare sparseness on distinct stimuli with sparseness to local variations, we compared the maximum sparseness across morph lines with the sparseness on the reference set after excluding that particular morph pair. This too yielded a strong positive correlation (*r* = 0.90, *P* < 0.000005; Fig. 3*B*). Indeed, sparseness values across all pairs of stimulus sets (reference stimuli or morph lines) were strongly correlated across neurons (Fig. 3*C*). We repeated this analysis with the average sparseness across all four morph lines and found similar correlations. Thus selective IT neurons respond to a few stimuli and are sharply tuned to local variations of these stimuli.

#### Does neuronal selectivity co-vary with waveform properties or recording location?

**Fig. 4.**
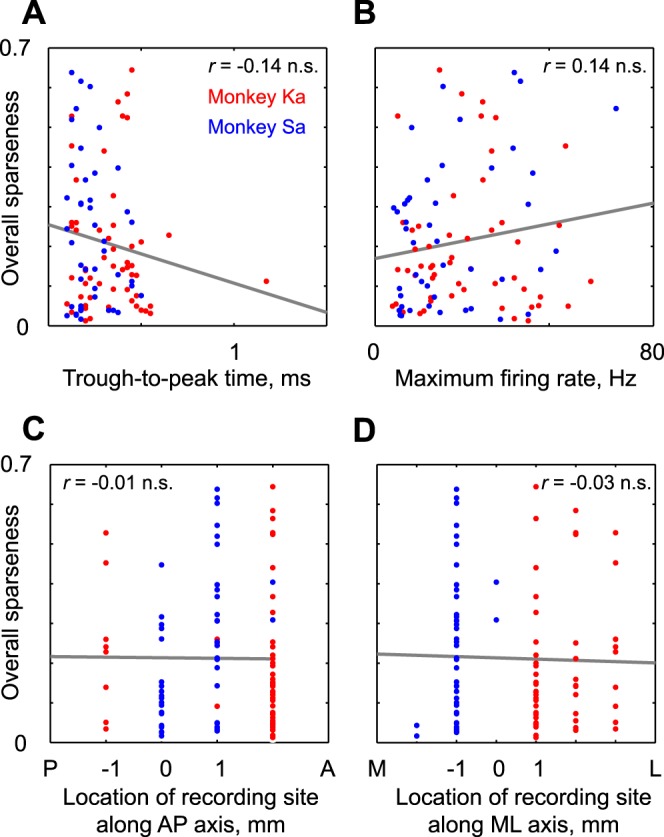
Relationship between sparseness and other intrinsic properties. *A*: overall sparseness for each neuron (on the entire stimulus set) plotted against the width of its action potential (peak-to-trough time). Red and blue dots correspond to neurons from monkey *Ka* and monkey *Sa*, respectively. The correlation coefficient is indicated in the plot (n.s., not statistically significant). *B*: overall sparseness for each neuron plotted against its maximum firing rate (across all stimuli). *C*: overall sparseness for each neuron plotted against its recording location along the anterior-posterior (AP) axis relative to the center of the recording chamber for each monkey. *D*: overall sparseness for each neuron plotted against its recording location along the medial-lateral (ML) axis relative to the center of the recording chamber for each monkey.

Next, we asked whether neuronal selectivity was correlated with various other intrinsic properties of each neuron. First, we asked whether selectivity of each neuron is correlated with the properties of its action potential. Figure 3*A* shows the waveforms of a few isolated units together with their sparseness. It can be seen that there is no obvious relationship between sparseness and the action potential shape. To investigate this further, we plotted the sparseness for each neuron (calculated across all stimuli) against the peak-to-trough width of its isolated action potential. This revealed no significant correlation (*r* = −0.14, *P* = 0.18; Fig. 4*A*). Second, we asked whether the sparseness of a neuron depends on its maximum firing rate. This too revealed no statistically significant correlation (*r* = 0.14, *P* = 0.17; Fig. 4*B*). Finally, we asked whether the sparseness of a neuron varied with its recording location in the cortex. We observed no significant correlation between sparseness of a neuron and its anterior-posterior recording location (*r* = −0.01, *P* = 0.96; Fig. 4*C*) or its medial-lateral location (*r* = −0.03, *P* = 0.77; Fig. 4*D*).

#### Do these results depend on the specific measure of neuronal tuning?

To confirm that the above correlations were not due to the specific measure of tuning used, we repeated the above analyses using another measure: for each neuron, we sorted stimuli from best to worst and identified the stimulus rank at which the response reached one-half of its maximum. This measure also yielded highly consistent correlations (correlation between average tuning widths measured along pairs of morph lines: *r* = 0.42, *P* < 0.05; correlation between reference set tuning width and morph tuning width: *r* = 0.47, *P* < 0.0005). For the morph line responses, we also measured neuronal selectivity using the rate of change in the response (estimated as the slope of the best fitting line). Here too, we observed a significant positive correlation between slopes along all morph lines (average correlation between morph line pairs: *r* = 0.60, *P* < 0.000005). Thus our results are not specific to a particular measure of neuronal selectivity.

#### Do these results depend on spontaneous activity levels across neurons?

To confirm that the above correlations did not depend on the spontaneous activity levels of each neuron, we repeated the above analyses after subtracting the spontaneous activity level for each neuron (estimated in a 200-ms window before the onset of the first stimulus in each trial). We obtained highly consistent correlations (average sparseness correlation between morph line pairs: *r* = 0.56, *P* < 0.000005; correlation between reference set sparseness and maximum morph line sparseness: *r* = 0.78, *P* < 0.000005). Thus our results are qualitatively similar even upon subtracting baseline activity from the neural response.

#### Are selective IT neurons also selective for identity-preserving changes?

**Fig. 5.**
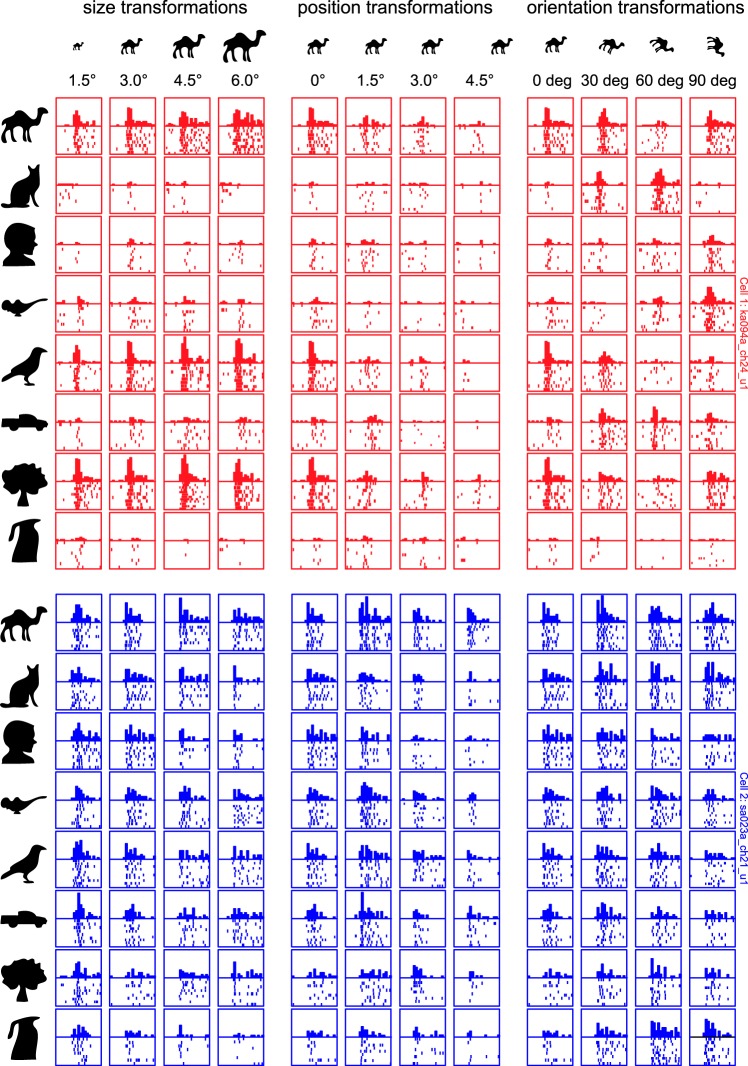
Example responses for size, position, and orientation variations. The neurons are the same as depicted in Fig. 2. To examine tolerance to identity-preserving transformations, each shape in the reference set was presented at 3 additional sizes, positions, and orientations. The sparse neuron (red rasters) shows greater modulation to changes in size, position, and orientation, whereas the distributed neuron (blue rasters) shows gradual changes. Conventions are as before.

If highly selective neurons are selective to variations along many dimensions, then are they also selective for variations along identity-preserving transformations? To test this possibility, we measured neural responses for each of the reference stimuli to variations in size, position, and orientation. The responses of the example neurons of Fig. 2 to these variations are shown in Fig. 5. The first neuron, which showed sparse responses to diverse stimuli, was also sharply tuned for size, position, and orientation. The second neuron, which showed distributed responses to the diverse stimuli, showed broad tuning for size, position, and orientation.

**Fig. 6.**
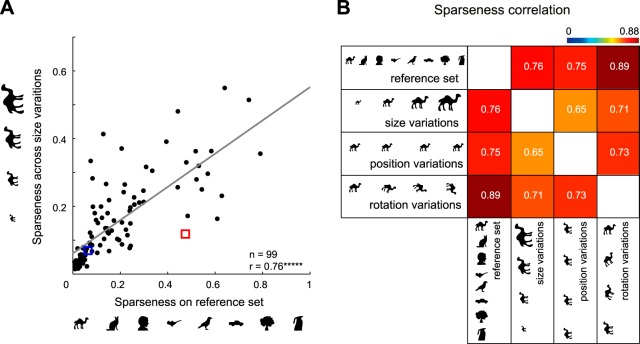
Highly selective neurons are also selective for size, position, and orientation. *A*: sparseness along size variations plotted against sparseness on the reference set for each neuron. *B*: matrix of pairwise correlations between sparseness on the reference set with average sparseness along size, position, and orientation variations. All correlations were statistically significant (*****P < 0.000005).

To examine these trends across the population, we calculated for each neuron the sparseness of responses to size variations of each shape and averaged this across shapes to obtain an average size sparseness for that neuron. We calculated analogous measures for position and orientation as well. We then asked whether sparseness on the diverse set would predict sparseness along these variations as well. We found a strong correlation between size sparseness and reference set sparseness (*r* = 0.76, *P* < 0.000005; Fig. 6*A*). In general, all sparseness pairs were strongly correlated (Fig. 6*B*). Thus highly selective neurons are also highly selective for changes in size, position, and orientation.

**Fig. 7.**
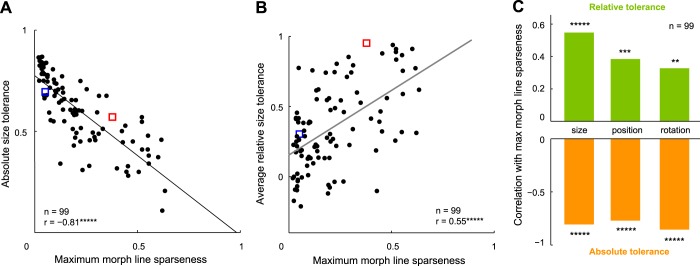
Relationship between tolerance and selectivity in IT neurons. *A*: absolute size tolerance for each neuron plotted against reference set sparseness. The absolute size tolerance is a measure of how much the neural response changes with changes in stimulus size (see materials and methods). *B*: relative size tolerance for each neuron plotted against reference set sparseness. Relative tolerance is a measure of how strongly a neuron maintains its shape preferences across changes in size (see materials and methods). *C*: summary of correlations of reference set sparseness with relative tolerance and absolute tolerance. **P < 0.005; ***P < 0.0005; *****P < 0.000005.

Previous studies have defined tolerance to an identity-preserving transformation as the degree to which a neuron maintains the same firing rate across changes in size, position, etc. (Zoccolan et al. 2007). By this definition, a neuron that is highly selective for size, position, or orientation will be less tolerant. Thus our finding is consistent with the observation of a tradeoff between selectivity and tolerance (Zoccolan et al. 2007). To be sure that these two results are essentially the same, we calculated for each neuron a measure of tolerance to changes in size, position, and orientation, akin to that used by the Zoccolan et al. (2007) study (see materials and methods). We calculated the absolute tolerance for each neuron separately for size, position, and orientation changes and asked how each measure varies with sparseness. For size tolerance, this revealed a significant negative correlation (r = −0.81, *P* < 0.000005; Fig. 7*A*). We found significant negative correlations for position and orientation changes as well (*r* = −0.77 and −0.85, respectively, *P* < 0.000005; Fig. 7*C*). These correlations were qualitatively similar for baseline-corrected firing rates as well (*r* = −0.46, −0.51, and −0.66, *P* < 0.000005, for size, position, and orientation, respectively).

#### Are selective IT neurons more invariant?

Although a neuron may be modulated strongly by stimulus size, its shape preference may remain unchanged at each size. This suggests an alternative measure of tolerance—which we denote as relative tolerance—that represents the degree to which the neuron maintains its shape preferences across changes in size (or likewise, for position and orientation). For each neuron, we calculated relative size tolerance for each pair of sizes as the correlation coefficient between the firing rates elicited by the eight reference shapes at the two sizes. The average relative size tolerance was then simply the relative tolerance averaged across all size pairs. We calculated analogous measures of relative tolerance for position and orientation. We then asked whether the maximum sparseness along morph lines is correlated with the average relative tolerance across neurons. For size changes, we observed a significant positive correlation between relative tolerance and sparseness (*r* = 0.55, *P* < 0.000005; Fig. 7*B*). This was also true for position and orientation changes (*r* = 0.39, *P* < 0.0003, for position; *r* = 0.33, *P* < 0.005, for orientation; Fig. 7*C*).

This correlation persisted even upon calculating the tolerance or sparseness measures for only size/position/orientation levels that elicited at least one significant visual response. To confirm that the above results were not due to the specific measure of relative tolerance used here, we repeated the analysis using a measure of separability of tuning that models the neuronal response as a product of tuning for shapes and tuning for size/position/rotation (Brincat and Connor 2004). This too revealed a positive correlation (*r* = 0.48, *P* < 0.000005, for size; 0.38, *P* < 0.0005, for position; and 0.34, *P* < 0.005, for orientation). We obtained similar results upon recalculating them using baseline-corrected firing rates (*r* = 0.34, *P* = 0.004, for size; *r* = 0.20, *P* = 0.05, for position; *r* = 0.34, *P* = 0.0005, for orientation).

Finally, we considered the possibility that the correlation between sparseness and relative tolerance might arise because sharply tuned neurons are likely to show greater relative tolerance simply because they produce a larger range of responses. To assess this possibility, we calculated for each neuron the consistency in its responses across the eight reference shapes for each transform level, using the correlation between the mean response derived from odd- and even-numbered trials. We took the maximum consistency across transform levels as an estimate of the maximum achievable correlation across size variations (and likewise, for position and orientation). We then divided the relative tolerance for each neuron by this estimate of consistency. If broadly tuned neurons were simply less-consistent due to a smaller range of responses, then normalizing the relative tolerance of each neuron by its consistency would result in similar relative tolerance estimates as for sharply tuned neurons. The resulting correlations between sparseness and normalized relative tolerance were smaller but remained statistically significant (*r* = 0.31, *P* < 0.005, for size; *r* = 0.26, *P* = 0.01, for position; *r* = 0.23, *P* = 0.02, for orientation).

We conclude that highly selective IT neurons are more invariant in the sense that they preserve their shape preferences across identity-preserving transformations.

#### Do IT neurons show correlated tuning for shape and texture?

Having established that neurons show correlated tuning across many shape dimensions, we wondered whether this would be true for properties other than shape. To address this question, we recorded the responses of 28 neurons in one monkey (monkey *Ka*) to two independent sets of stimuli: one containing 80 diverse silhouette shapes and the other containing 480 natural textures. All aspects of experiment design were identical to the present study except for the stimuli.

**Fig. 8.**
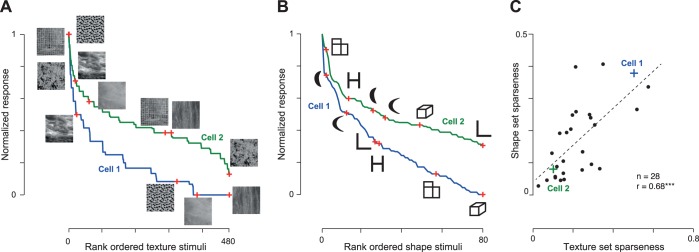
Selectivity for shape and texture across neurons. *A*: example responses for 2 IT neurons to a set of natural textures. The normalized responses of each cell are rank ordered from best to worst, with sample stimuli shown along the tuning curve. Blue curve, first neuron; green curve, second neuron. *B*: example responses for the same 2 neurons to a set of silhouette shapes. *C*: sparseness for the shape set plotted against the sparseness of the texture set across the sampled neural population (*n* = 28). The example neurons are highlighted using crosses. ***P < 0.0005.

The responses of two example cells to the texture set (Fig. 8*A*) and to the shape set (Fig. 8*B*) illustrate the general trend we observed across the population. The first neuron was sharply tuned to the texture set and the shape set, whereas the second neuron was broadly tuned to both sets. To investigate this pattern across the population, we calculated sparseness for each neuron for each set on its firing rates calculated in a window 50–200 ms after stimulus onset. This revealed a significant positive correlation (*r* = 0.68, *P* < 0.0005; Fig. 8*C*). This correlation remained significant even when sparseness was calculated on baseline-corrected firing rates (*r* = 0.42, *P* < 0.05). It also remained significant when the sparseness for both sets was calculated on equal numbers of stimuli (*r* = 0.67, *P* < 0.0005; on average, between sparseness computed for each neuron on 80 randomly chosen textures and 80 shapes). We conclude that neurons that are sharply tuned for texture are also sharply tuned for shape.

## DISCUSSION

The main finding of our study is that selective IT neurons are selective along many dimensions. This finding implies that there is an intrinsic, dimensionality-reducing constraint on tuning in IT. We have shown that selective IT neurons respond to fewer stimuli and are narrowly tuned along a number of shape dimensions (variations along morph lines or along size, position, and orientation changes) and even independent dimensions, such as shape and texture. A second finding is that selective IT neurons are also more invariant in the sense that they prefer the same shape across changes in size, position, and orientation. Below, we discuss the implications of our results in the context of the existing literature.

### 

#### Relation to studies of tuning in visual cortex.

Our finding that IT neurons are highly selective along many stimulus dimensions is similar to reports in primary visual cortex (V1) that spatial frequency bandwidth and orientation bandwidth are correlated (De Valois et al. 1982; Stevens 2004; Xing et al. 2004). We propose that strength of tuning is an intrinsic property for a neuron, independent of its feature tuning, and this is likely true throughout visual cortex. What would make one neuron generally more selective and another less? One possibility is that more-selective neurons differ in their action potential properties, such as threshold. However, we observed no systematic correlation between sparseness and action potential shape (Fig. 3*A*). Although there are differences in selectivity between excitatory and inhibitory neurons (Mruczek and Sheinberg 2012), we did not observe any clear clustering of waveforms into these putative cell types, possibly because there were no inhibitory neurons in our recorded population. The other possibility is that selective neurons either have selective inputs or stronger local inhibitory interactions. It has been shown in simulation that local inhibitory interactions can cause V1 neurons to become selective along multiple feature dimensions (Xing et al. 2004; Zhu et al. 2010). These possibilities will require further study.

Our finding, however, stands in disagreement with an earlier study, where IT neurons were tested using shapes that varied in dimensions, such as curvature and aspect ratio of rectangles (Kayaert et al. 2005). In this study, neurons showed no clear correlation between their modulation along these dimensions [cf. Fig. 10 in Kayaert et al. (2005)]. This discrepancy may be due to the fact that there were large variations in response modulation along one of the shape dimensions across neurons (typically, curvature) and relatively small variations along another (typically, aspect ratio or taper), resulting in little or no tuning correlation. In contrast, in our study, we have used morph lines between distinct stimuli (differing along multiple features) that may have resulted in larger response modulation, revealing the underlying correlation. The reconciliation of these two observations will require testing the same set of neurons along a larger range of curvature variations, as well as along morph lines between arbitrary shapes.

#### Selectivity-tolerance tradeoff vs. intrinsic tuning.

We have found that highly selective IT neurons are highly selective along many stimulus variations. Our findings represent a generalization of the observation that there is a tradeoff between selectivity and tolerance in IT and V4 (Rust and Dicarlo 2012; Zoccolan et al. 2007). In these studies, there is a negative correlation between shape selectivity and tolerance (as defined by the lack of modulation to size and position). It can be readily seen that a neuron with low tolerance defined in this manner would be highly selective. Indeed, for the same data that showed a positive correlation between shape selectivity and size/position selectivity (Fig. 6), we found a negative correlation between selectivity and absolute tolerance (Fig. 7). However, we have made the important additional observation that selective IT neurons are not only selective to position/size/rotation but also selective to parametric variations along several distinct morph lines in the neighborhood of a variety of stimuli (Fig. 6). Taken together, these observations indicate that selective IT neurons are sharply tuned along all stimulus variations, not just along variations in size, position, etc. Thus rather than a tradeoff between selectivity and tolerance, our finding implies that there is an intrinsic component to neuronal tuning.

#### Selective IT neurons are also more invariant.

Our results further contradict the idea of a tradeoff between selectivity and tolerance in IT neurons by showing that selectivity and tolerance are, in fact, positively correlated (Fig. 7, *B* and *C*), when tolerance is defined as the degree to which the neuron preserves its shape preferences across changes in size/position/orientation. Indeed, many early studies have used this idea of invariance to claim that IT neurons show invariance across identity-preserving transformations (Brincat and Connor 2004; Ito et al. 1995; Sáry et al. 1993; Schwartz et al. 1983; Zoccolan et al. 2007). However, none of these studies have compared relative tolerance with shape selectivity. Our results show that selective neurons are also more invariant in that they preserve their shape preferences across identity-preserving transformations.

#### Implications for shape coding in IT neurons.

Our finding that selective IT neurons are selective along all stimulus variations has important implications for understanding their shape tuning. It is widely believed that neural responses in IT are complex tuning functions that depend on the underlying feature tuning. Our results show that there is an intrinsic constraint on the sharpness of tuning for the features coded by each IT neuron, making it always sharply tuned or always broadly tuned along all dimensions. In other words, the tuning functions of IT neurons are complex but contain systematic dependencies that constrain their dimensionality. Our results may constrain biologically plausible models of vision by requiring selective neurons to remain selective everywhere. Finally, why would there be such an organization at all in visual cortex? We speculate that the presence of highly selective neurons tuned along all feature dimensions implies that the same neuronal population can be modulated for a variety of tasks without the need for feature-specific gating. Whether this organization confers other specific advantages for object recognition will require further study.

## GRANTS

Funding for this research was provided by an Intermediate Fellowship from the Wellcome Trust-Department of Biotechnology (DBT) India Alliance and by the DBT-Indian Institute of Science (IISc) Partnership Programme (to S. P. Arun).

## DISCLOSURES

No conflicts of interest, financial or otherwise, are declared by the authors.

## AUTHOR CONTRIBUTIONS

Author contributions: K.A.Z. and S.P.A. conception and design of research; K.A.Z. and S.P.A. performed experiments; K.A.Z. and S.P.A. analyzed data; K.A.Z. and S.P.A. interpreted results of experiments; K.A.Z. and S.P.A. prepared figures; S.P.A. drafted manuscript; K.A.Z. and S.P.A. edited and revised manuscript; K.A.Z. and S.P.A. approved final version of manuscript.
